# Supplementary material for: Cln3 can work independently of Whi5 on the cell size for Start in yeast
Source: bioRxiv. 2025 Oct 25:2025.10.24.684447. Preprint. [Version 1] doi: 10.1101/2025.10.24.684447 (PMC12633300; doi:10.1101/2025.10.24.684447)
Supplement: 1 [file NIHPP2025.10.24.684447V1-supplement-1.pdf]

bioRxiv preprint doi: https://doi.org/10.1101/2025.10.24.684447; this version posted October 25, 2025. The copyright holder for this preprint (which was not certified by peer review) is the author/funder, who has granted bioRxiv a license to display the preprint in perpetuity. It is made available under aCC-BY 4.0 International license.

Supplementary Tables S1 and S2

Table S1. Cell sizes in the GZ background, in fL.

**Background: GZ238/GZ239**						
N	Genotype	Mean (SE)	Median (SE)	Mode (SE)	C.V.	Strain
6	*CLN3 WHI5*	50.6(0.06)	45.3 (0.00)	35.1 (0.89)	0.46	AS-9D
6	*CLN3* ***whi5***	46.6(0.34)	40.6 (0.25)	29.7 (0.51)	0.54	AS-9C
6	***cln3*** *WHI5*	76.1(0.32)	69.5 (0.32)	54.7 (1.41)	0.44	AS-9A
6	***cln3 whi5***	57.8(0.18)	51.2 (0.00)	38.3 (0.91)	0.48	AS-9B
6	*CLN3 WHI5*	49.5 (0.26)	44.8 (0.32)	34.4 (0.91)	0.46	AS-10A
6	*CLN3* ***whi5***	45.5 (0.20)	39.3 (0.00)	28.7 (0.44)	0.54	AS-10D
6	***cln3*** *WHI5*	76.4 (0.33)	69.8 (0.34)	58.6 (1.59)	0.44	AS-10C
6	***cln3 whi5***	56.6 (0.63)	50.5 (0.51)	38.8 (0.73)	0.48	AS-10B
6	*CLN3 WHI5*	49.2 (0.32)	43.8 (0.00)	34.1 (0.99)	0.49	AS-12B
6	*CLN3* ***whi5***	45.8 (0.18)	39.6 (0.25)	30.2 (0.80)	0.53	AS-12A
6	***cln3*** *WHI5*	74.4 (0.41)	67.8 (0.46)	52.4 (2.64)	0.44	AS-12C
6	***cln3 whi5***	55.4 (0.21)	48.2 (0.00)	37.4 (1.05)	0.50	AS-12D
8	Diploid	89.2 (0.38)	83.6 (0.33)	66.6 (0.96)	0.37	Diploid

bioRxiv preprint doi: https://doi.org/10.1101/2025.10.24.684447; this version posted October 25, 2025. The copyright holder for this preprint (which was not certified by peer review) is the author/funder, who has granted bioRxiv a license to display the preprint in perpetuity. It is made available under aCC-BY 4.0 International license.

Table S2. Cell sizes in the BY background, in fL.

**Background: BY4741/BY4742**						
N	Genotype	Mean	Median	Mode	C.V.	Strain
1	*CLN3 WHI5*	38.4	35.7	29.9	0.39	YSH 675-2A
1	*CLN3* ***whi5***	30.4	27.8	24.2	0.41	YSH 675-2D
1	***cln3*** *WHI5*	61.2	55.9	43.0	0.42	YSH 675-2C
1	***cln3 whi5***	43.4	40.0	32.8	0.41	YSH 675-2B
1	*CLN3 WHI5*	38.1	35.7	31.4	0.39	YSH 675-5C
1	*CLN3* ***whi5***	33.4	30.7	24.9	0.43	YSH 675-5D
1	***cln3*** *WHI5*	64.2	58.8	49.4	0.44	YSH 675-5A
1	***cln3 whi5***	40.6	37.2	30.7	0.44	YSH 675-5B
1	*CLN3 WHI5*	37.2	35.0	29.9	0.38	YSH 675-6A
1	*CLN3* ***whi5***	30.4	28.5	25.6	0.39	YSH 675-6B
1	***cln3*** *WHI5*	65.0	59.5	46.5	0.44	YSH 675-6D
1	***cln3 whi5***	41.4	38.6	31.4	0.40	YSH 675-6C
